# The Graphene Squeeze-Film Microphone

**DOI:** 10.1021/acs.nanolett.4c02803

**Published:** 2024-11-04

**Authors:** Marnix P. Abrahams, Jorge Martinez, Peter G. Steeneken, Gerard J. Verbiest

**Affiliations:** † Department of Precision and Microsystems Engineering, Delft University of Technology, Mekelweg 2, 2628 CD Delft, The Netherlands; ‡ Multimedia Computing Group, Intelligent Systems Department, Faculty of Electrical Engineering, Mathematics and Computer Science, Delft University of Technology, 2628 XE Delft, The Netherlands

**Keywords:** graphene, microphone, squeeze-film effect, membrane, resonance frequency, gas pressure

## Abstract

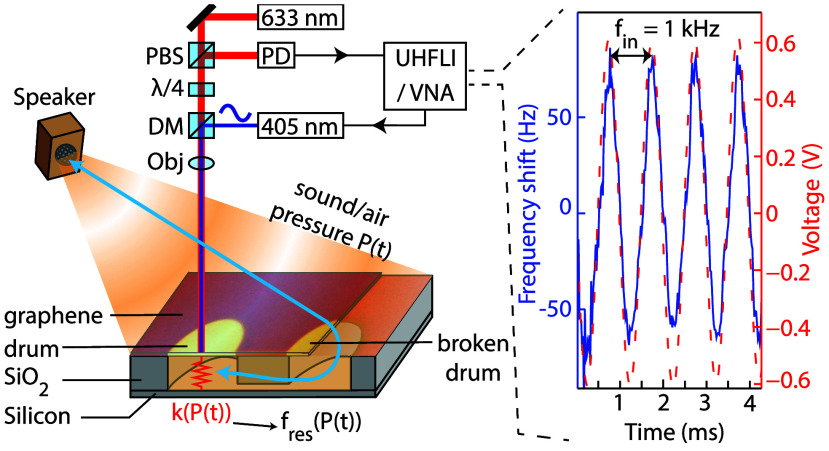

Most microphones detect sound-pressure-induced motion of a membrane. In contrast, we introduce a microphone that operates by monitoring sound-pressure-induced modulation of the air compressibility. By driving a graphene membrane at resonance, the gas, that is trapped in a squeeze-film beneath it, is compressed at high frequency. Since the gas-film stiffness depends on the air pressure, the resonance frequency of the graphene is modulated by variations in sound pressure. We demonstrate that this squeeze-film microphone principle can be used to detect sound and music by tracking the membrane’s resonance frequency using a phase-locked loop. The squeeze-film microphone potentially offers advantages like increased dynamic range, lower susceptibility to pressure-induced failure and vibration-induced noise over conventional devices. Moreover, microphones might become much smaller, as demonstrated in this work with one that operates using a circular graphene membrane with an area that is more than 1000 times smaller than that of MEMS microphones.

During the last centuries, a variety of membrane based devices have been developed to detect sound and pressure.^1−3^ In static pressure sensors (Figure 1a) the membrane deflection is a measure of the difference between the outside pressure *P*_amb_ and the gas pressure *P*_gas_ in a sealed reference cavity. Condenser microphones (Figure 1b) operate by a similar principle, however they contain a small perforation, which causes static pressure differences to equilibriate at a time constant τ_eq_, such that they only respond to pressure variations at sound frequencies *f*_s_ > 1/τ_eq_.

**Figure 1:**
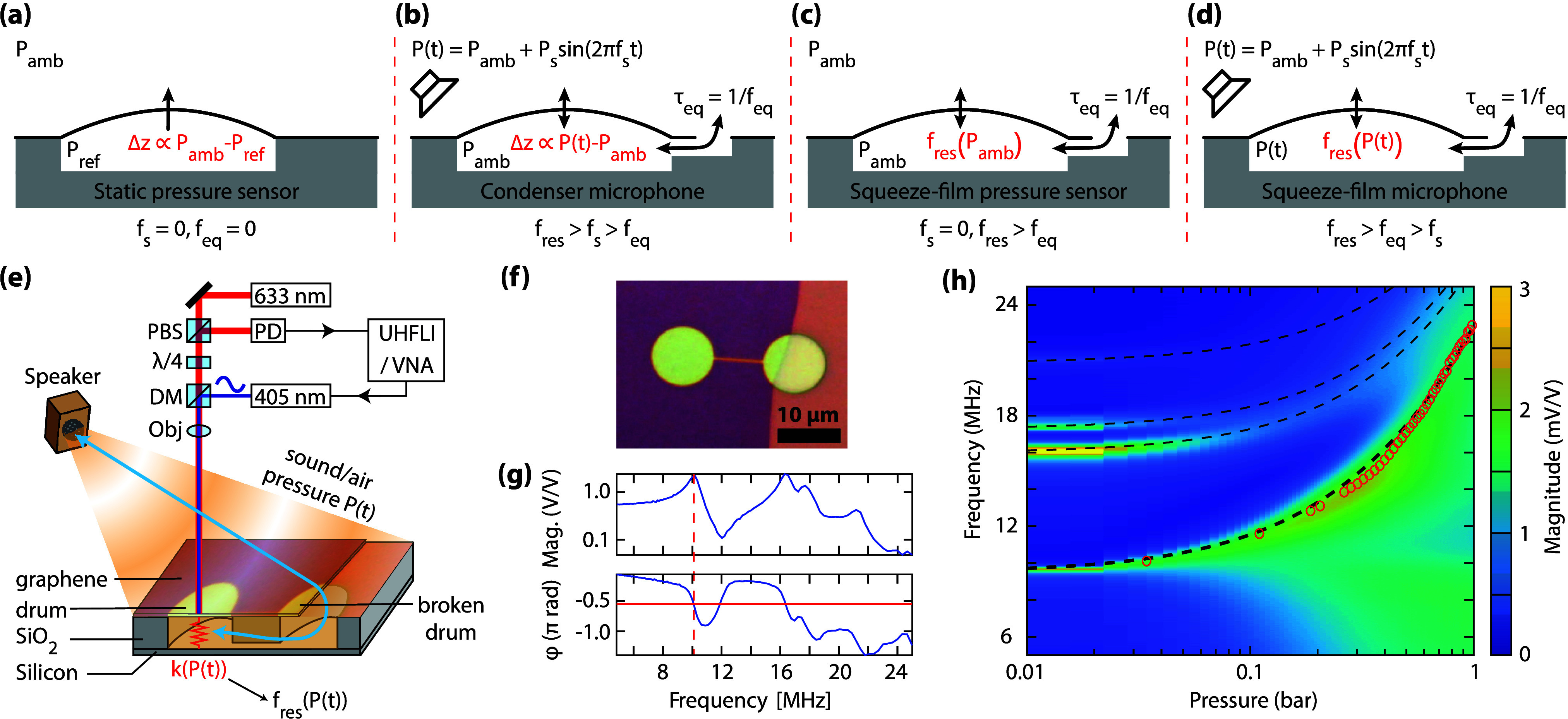
Schematic drawings of (a) static pressure sensor, (b) condenser microphone, (c) squeeze-film pressure sensor, and (d) squeeze-film microphone. The squeeze-film devices distinguish them from conventional devices by having a pressure below the membrane that is always equal to the external pressure, caused by the relatively short equilibration time τ_eq_. Instead of measuring pressure by monitoring the deflection Δ*z* of the membrane, the squeeze-film devices determine gas pressure by monitoring its effect on the membrane’s resonance frequency *f*_res_. (e) Illustration of a graphene drum over a cavity in a SiO_2_/Si substrate. A red (633 nm) laser beam passes a polarized beam splitter (PBS) and a quarter-wave plate (λ/4) such that the reflected beam is diverted into a photodetector (PD) for analysis by a phase-locked loop (PLL, implemented in UHFLI) or vector network analyzer (VNA). A blue (405 nm) laser beam enters the red laser beam path through a dichroic mirror (DM) and excites the drum at a given frequency. The objective (Obj) focuses both laser beams on the drum. A speaker modulates the air pressure *P*(*t*) by sound waves, which in turn modulate the spring constant *k* of the drum and thus its resonance frequency *f*_res_. (f) An optical image of a typical device shows a suspended drum that is connected to the environment by a venting channel. (g) The magnitude and phase as a function of frequency measured by the VNA at a pressure of 10 mbar. The continuous red line indicates the −π/2 phase shift at the resonance frequency indicated by the dashed red line. (h) Magnitude as a function of frequency and pressure as recorded with the VNA. Red circles represent the extracted resonance frequencies and the black dashed lines fits to eq 1.

A more recent concept is the squeeze-film pressure sensor^4,5^ (Figure 1c), which consists of a membrane that is separated from a back-plate by a narrow gap with thickness *g*_0_ that contains a thin film of gas which is at the same average pressure *P*_amb_ as the surrounding gas. The effective stiffness *k*_eff_ of the membrane does not only depend on its mechanical properties, but also on the compressibility of the gas. Since this compressibility is pressure dependent, the sensor’s resonance frequency , in which *m*_eff_ is the effective mass of the membrane, depends on pressure according to the following equation^6^
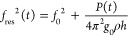
1where ρ is the membrane’s mass density, *h* its thickness, and *f*_0_ its resonance frequency in vacuum.

Here we introduce and investigate a microphone that detects sound using the squeeze-film effect (Figure 1d), which we also patented.^7^ The operation principle of the squeeze-film microphone is based on the use of eq 1 to determine the time-dependent sound pressure *P*_s_(*t*). This requires monitoring the resonance frequency *f*_res_(*t*) at the sound frequency *f*_s_. As shown in Figure 1d, when sound modulates the gas pressure as *P*(*t*) = *P*_amb_ + *P*_s_ sin(2*πf*_s_*t*), the time-dependence of the resonance frequency is found to be
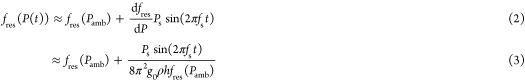
2For the squeeze-film microphone to detect sound pressure variations, the membrane needs to leak gas by design^8^ such that the time τ_eq_ = 1/*f*_eq_ it takes for the pressure in the squeeze-film to equilibrate with the ambient gas is much shorter than the period of the sound τ_s_ = 1/*f*_s_. Also, for the squeeze-film gas to have a substantial effect on the resonance frequency, the period of the sound needs to be long compared to the typical response time *Q*/*f*_res_ of the resonator, which is less than 1 μs for the devices under investigation. Combining these conditions, we find that we need to design the squeeze-film microphone to satisfy *f*_s_ < *f*_eq_ < *f*_res_/*Q*.

Graphene is the ideal material to satisfy these conditions, especially because of its low mass per unit area and high flexibility, which typically results in high resonance frequencies *f*_res_ ≈ 10 MHz for membranes with a diameter of 10 μm and a *Q*-factor of ≈3 in ambient conditions and ≈150 in vacuum conditions.^9,10^ The time it takes to equilibrate the pressure below the membrane via a channel is typically^8^ in the range of τ_eq_ = 1–10 μs, corresponding to *f*_eq_ = 100–1000 kHz, while the audible spectrum is in the range of *f*_s_ = 20 Hz to 20 kHz. Moreover, eq 1 shows that the low mass per area *ρh* of graphene results in a high sensitivity of the resonance frequency to pressure variations, especially if the gap *g*_0_ is small.

To realize a squeeze-film microphone (Figure 1f), we exfoliate few-layer graphene over a *g*_0_ = 285 nm deep dumbbell cavity with a radius of 5 μm in SiO_2_ (see details in Supporting Information 1). In total we measured six graphene squeeze-film microphones. We characterize the devices in a photothermal setup,^11,12^ in which an intensity modulated blue laser (λ = 405 nm) generates a thermal expansion force on the graphene drum near its resonance frequency and a red laser (λ = 633 nm) measures its motion (Figure 1e). For each device, we measure the membrane’s frequency response (Figure 1g) as a function of air pressure at room temperature to confirm the behavior predicted by eq 1. We extract the resonance frequency from experimental data at a given pressure, see dashed red line in Figure 1g and dashed lines in Figure 1h. The slope of the resonance frequency versus pressure at 1.0 bar (Figure 1h), defines the sensitivity  to sound pressure waves. By taking the pressure derivative of eq 1 this sensitivity is found to be *S*_f_ = (8π^2^*f*_res_*g*_0_*ρh*)^−1^. From the experimental data we obtain a typical sensitivity of *S*_f_ = 200 Hz/Pa, which is well within 10% of the theoretical prediction. From this value we find that a squeeze-film microphone can capture a normal conversation, which corresponds to a root-mean-square (rms) sound pressure level of about 0.04 Pa (=65 dB_SPL_), if it can detect resonance frequency modulations of ∼8 Hz.

After determining the pressure sensitivity of the resonance frequency, we use a Phase-Locked-Loop (PLL, Zurich Instruments UHFLI^13^) to track the resonance frequency of the graphene membrane as a function of time. We continuously update the frequency of the intensity modulation of the blue laser with the tracked resonance frequency to keep driving the graphene membrane at its resonance frequency. Then, we generate sinusoidal sound waves at frequency *f*_in_ and sound pressure level ∼100 dB_SPL_ = 2.0 Pa at the location of the microphone with a speaker. We record the sound both with a reference microphone and with the graphene squeeze-film microphone. Figure 2a shows an exemplary measurement at *f*_in_ = 1 kHz. The blue curve in the figure (left axis) shows the resonance frequency shift of the graphene microphone with respect to the nominal value of ∼22 MHz, due to the squeeze-film effect as determined by the PLL, while the red dashed line (right axis) shows the corresponding signal detected by the reference microphone. The correspondence between both signals provides evidence of the functionality of the squeeze-film microphone. We note that the response is somewhat lower than expected from the sensitivity of 200 Hz/Pa as estimated from eq 1, which we attribute to the condition *f*_res_/*Q* > *f*_eq_ > *f*_s_ that may not be fully obeyed at 1 kHz and to the limitations of the PLL.

**Figure 2:**
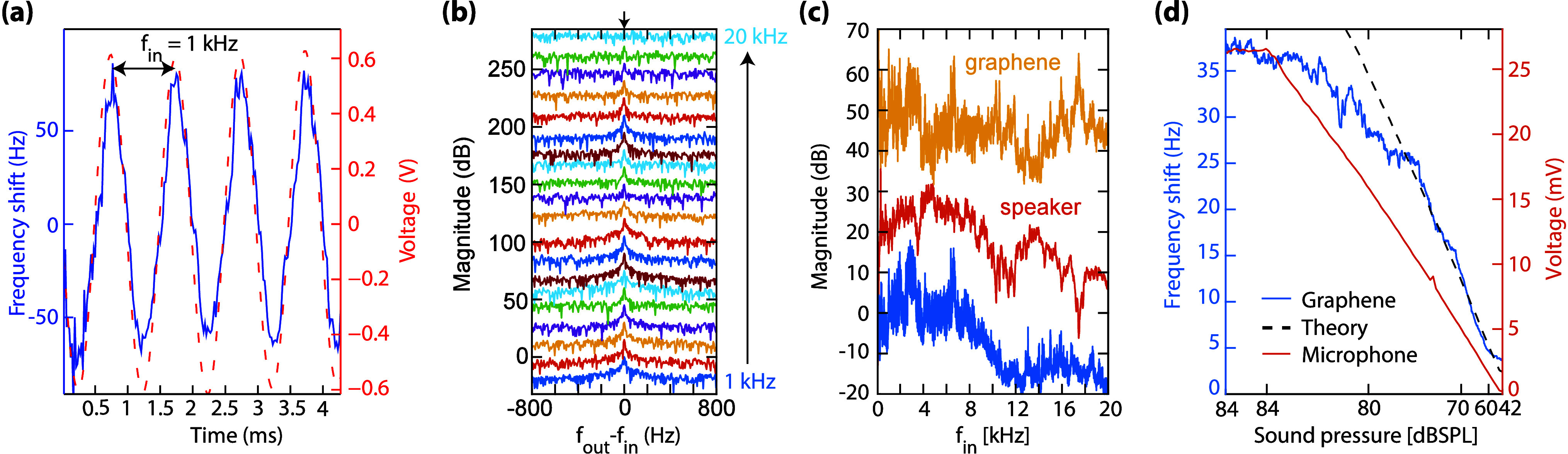
(a) A time-snapshot of the shift in resonance frequency of the drum (blue) and the output of the reference microphone (red) due to a 1 kHz sound wave at 100 dB_SPL_. (b) Waterfall plot of typical spectral responses of the drum around frequencies *f*_out_ = *f*_in_ with *f*_in_ ranging from 1 kHz to 20 kHz. All curves are offsetted by 15 dB from one another. (c) The raw spectral response of the drum (blue) and of the speaker (red). After compensating for the speaker response, the graphene drum (yellow) shows a flat response for all audible frequencies. All curves are offsetted by 25 dB from one another. In panels (b) and (c), the dB-scale is obtained by using the amplitude of 68 Hz received at *f*_in_ = 1 kHz and 100 dB_SPL_ (see panel (a)) as reference. (d) Response of the graphene drum (blue) and reference microphone (red) to a 1 kHz sound wave for sound pressures ranging from 84 dB_SPL_ to 42 dB_SPL_. The black line indicates the response expected from eq 1.

We repeat the measurement at frequencies *f*_in_ ranging from 1 kHz to 20 kHz and take the Fourier transform of the time signal coming from the PLL, like that in Figure 2a, to obtain the spectral responses depicted in Figure 2b. The spectra show a clear response of the graphene squeeze-film microphone at the input frequency *f*_out_ = *f*_in_. The observed reduction of its output at 20 kHz can mainly be attributed to a reduction of the output power of the speaker at high frequencies (see Figure 2c). To make sure the detected sound signal is transmitted via air, and not generated by vibrations of the support of the microphone, we mounted the graphene devices on a vibrating piezo-electric transducer.^14^ By actuating the transducer at the membrane resonance frequency, we were not able to detect the motion and resonance of the membrane in air, providing evidence that the detected sound signal does not propagate through the sample support or substrate, but is transmitted via air (Supporting Figure 1). By comparing the output of the graphene squeeze-film microphone to that of a calibrated reference microphone, we can obtain its transfer function, that is shown in Figure 2c. How we determined the transfer functions of the reference microphone and speaker is provided in Supporting Information 2. As Figure 2c indicates and expected based on eq 1, the sensitivity of the graphene membrane is roughly constant over the probed acoustic frequency range.

To determine the minimally detectable sound pressure level, we generate sinusoidal sound waves at frequency *f*_in_ = 1 kHz and a strength varying from 84 dB_SPL_ to 42 dB_SPL_. Figure 2d shows that the response of the graphene device agrees nicely with the one expected based on eq 1. Moreover, it follows the signal measured by the reference microphone quite well. Graphene devices can thus sense sound pressure levels down to 42 dB_SPL_.

The presented graphene devices even allow for the recording of music. A 5.5 s long spectrogram of a song^15^ recorded with a graphene device and with the reference microphone is shown in Figure 3a and 3b, respectively. Full recordings are provided on Zenodo.^15^ By comparing both panels of Figure 3, we observe the response of the graphene device can captures the main features of the sound signal below ∼750 Hz, although it contains more noise than the reference microphone. The main sources of noise are probably (i) limitations of the PLL and (ii) the resonance frequency fluctuations of the membrane, which tend to increase for small device mass.^16^ Overall Figure 3 shows the functionality of graphene membranes with a diameter of only 10 μm as microphone.

**Figure 3:**
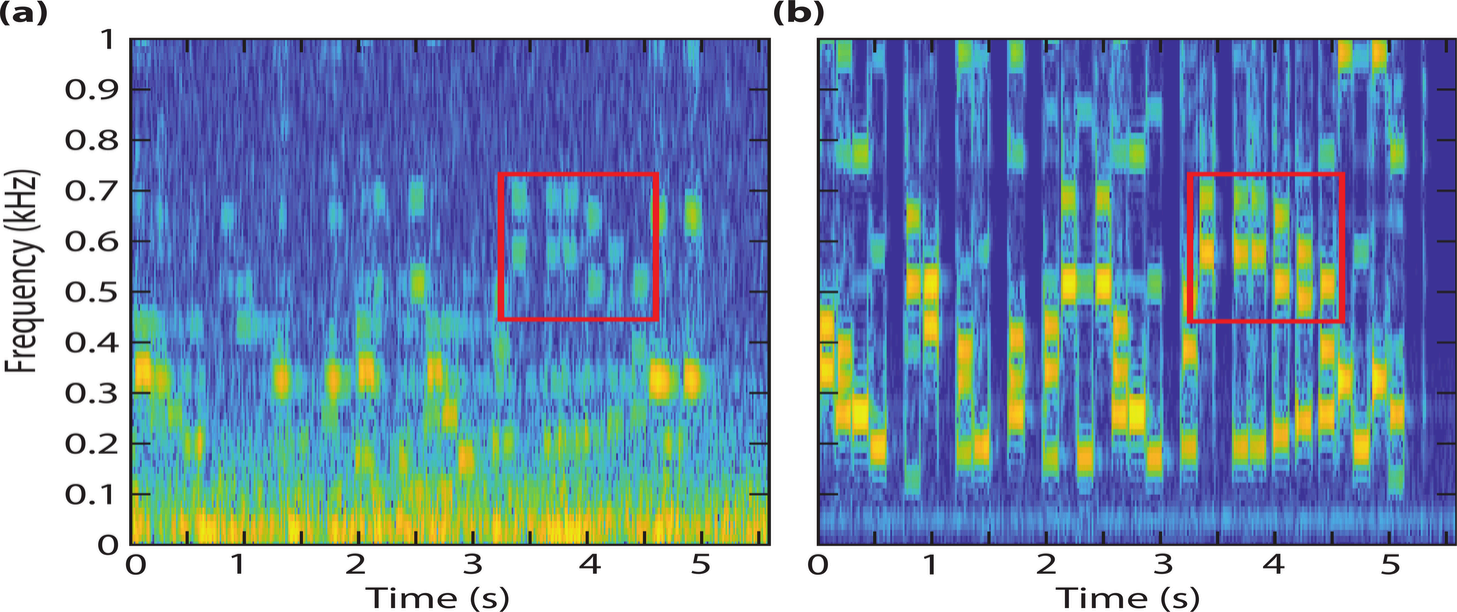
Selected normalized spectrograms of a song^15^ recorded with (a) the graphene squeeze-film microphone and (b) the reference microphone. The red squares enable direct comparison between data in panels (a) and (b).

To benchmark the fabricated graphene devices, we determine the signal-to-noise ratio (SNR) at 1 kHz according to the procedure that is used for commercial microphones. This process is explained in detail in Supporting Information 3. In short, a continuous sound wave of 1 kHz at 80 dB_SPL_ excites the microphone. For 5 different devices, the graphene microphone response (resonance frequency shift as a function of time) is recorded via the PLL and converted to the frequency domain using a Fourier transform. The signal strength is determined over a small bandwidth around 1 kHz, while the noise floor is also detected, like in Figure 2b. We also extract the strength of all harmonics of 1 kHz up to 20 kHz to determine the total harmonic distortion (THD). The rest of the spectrum is considered to be noise. The extracted SNR and THD values are listed in Figures 4a and 4b for all measured devices as a function of their resonance frequency *f*_res_ at atmospheric pressure, which varied due to tension and membrane thickness variations. As expected (Supporting Information 4, eq S7), the SNR increases proportional to the resonance frequency *f*_0_ up to a maximum of 23 dB, which corresponds to a detection limit of 80–23 = 57 dB_SPL_. With increasing *f*_res_, also the THD decreases to a minimum of 12%.

**Figure 4:**
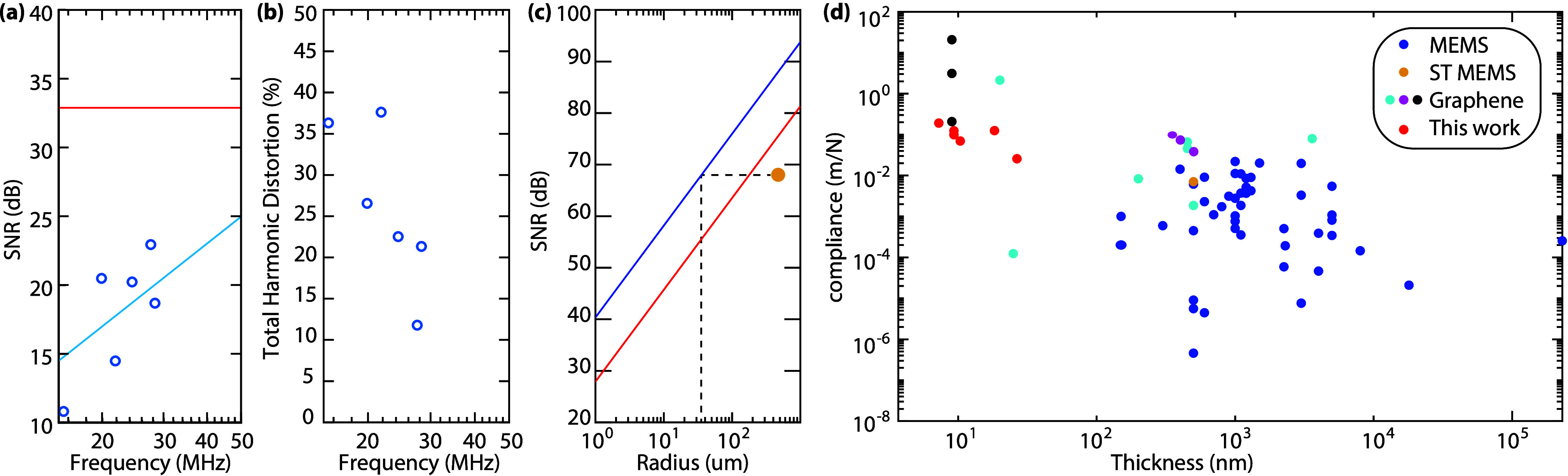
(a) Signal-to-noise ratio and (b) total harmonic distortion of the measured squeeze-film microphones (cyan circles) as a function of their resonance frequency. The SNR increases approximately linearly with the logarithm of the resonance frequency (cyan line in a). The red line in a indicates the SNR limit by thermo-mechanical noise. (c) Thermo-mechanical SNR limit at a sound pressure level of 80 dB (red) and 94 dB (blue). The orange point marks the performance of the MP23DB01HP of STMicroelectronics. The black dashed lines indicate the possible miniaturization by squeeze-film microphones at constant SNR. (d) Comparison of the mechanical compliance and thickness of the presented squeeze-film microphones (red) to other MEMS microphones (blue),^2^ to the MP23DB01HP of STMicroelectronics (orange), and different types of graphene microphones (cyan,^18−22^ magenta,^23−25^ black^26^). Panel (d) is adapted from ref (26). Available under a CC-BY 3.0. Copyright 2023 RSC Publishing.

The ultimate signal-to-noise ratio of the microphone is set by the thermo-mechanical motion of the graphene membrane. The squeeze-film effect translates this thermo-mechanical motion into frequency noise. As the signal is determined by eq 1 and the strength of the incoming pressure wave, the signal-to-noise ratio can be analytically computed (Supporting Information 4) and depends on the radius of the membrane, temperature, and ambient pressure. The outcome, for an incident sound pressure level of 80 dB_SPL_, is indicated by the red line in Figure 4a. The squeeze-film microphones presented in this work are roughly 20 dB below the thermo-mechanical SNR limit (blue circles in Figure 4a), which might be attributed to the contribution of other sources and frequency fluctuations than thermo-mechanical (Brownian) noise. To show the potential of squeeze-film microphones, we show the calculated (eq S8) thermo-mechanically limited SNR in Figure 4c for a sound pressure level of 80 dB (red) and 94 dB (blue) together with the specified SNR of the MP23DB01HP of STMicroelectronics^17^ for 94 dB_SPL_ at 1 kHz. The MP23DB01HP uses a membrane radius of 475 μm to reach this SNR level. However, a squeeze-film microphone reaches a (thermomechanical noise limited) SNR value of 64 dB at ambient pressure and room temperature at an almost 15x smaller radius of 18 μm. The experimental total harmonic distortion (see SI 3) of the devices under study is shown in Figure 4b.

The sensitivity of conventional MEMS microphones is determined by the compliance and thickness of the membrane. A high compliance, set by a low pretension in the membrane,^26^ and small thickness is desired for best microphone performance. For the squeeze-film microphone, this does not hold. In the thermo-mechanical limit, both the signal and noise scale identically with the membrane thickness and compliance. Consequently, the achievable SNR does not directly depend on the compliance nor on the thickness. To reach the thermo-mechanical limit, we just require a high resonance frequency to maintain the condition *f*_res_ > *f*_eq_ > *f*_s_. This could be achieved by increasing the membrane tension or decreasing the membrane mass per area (e.g., by reducing the membrane thickness). This lifts design and fabrication constraints, as high tension membranes can be routinely fabricated in Si_3_N_4_ technology and graphene microphones can also be fabricated on wafer-scale.^27^ Nevertheless, a high compliance can still be helpful, since although it does not affect the absolute sensitivity, it does improve the fractional sensitivity *S*_f_/*f*_res_. In that respect the high compliance values obtained with the graphene membranes (see Figure 4d) are beneficial.

Compared to conventional microphones the squeeze-film pressure sensor has several advantages. First of all, theoretically its sensitivity is approximately frequency independent and only depends on the gap size *g*_0_, the membrane size, tension, mass and dimensions according to eq S6. This means that very low sound frequencies can be detected, and the microphone can even operate simultaneously as static pressure sensor by monitoring *f*_res_. The maximum sound frequency detectable by the squeeze film is limited by the frequency *f*_eq_ which can be of the order of 100–1000 kHz. The squeeze-film microphone might therefore also operate in the ultrasound regime, although it will become more challenging to track the resonance fast enough in this range. Further potential advantages of the squeeze-film microphone are its relative robustness against sudden external pressure changes, due to its short pressure equilibration time τ_eq_ and its intrinsic flatband response, independent of frequency, based on eq 1. Moreover, we expect the squeeze-film microphone to be extremely robust at very high sound pressure levels, because the sound pressure does not directly result in a force on the membrane like in conventional microphones. On the other hand, a main challenge in the operation of the squeeze-film microphone is its relatively complex readout methodology. It will require advanced circuit design developments to be able to realize a readout circuit that can compete with the low-power, low-cost CMOS circuits that are used to readout current MEMS microphones.^28^

In summary, we present a new microphone concept utilizing the squeeze-film effect for detecting sound. We show that the microphone’s resonance frequency is indeed quite sensitive to pressure changes, and that the sound-induced frequency changes due to the tension modulation can be detected by a PLL circuit. The low mass and high-flexibility of graphene make it very suitable for this type of pressure sensing. Although the readout of the microphone is more complex, it offers several advantages like broad bandwidth, small membrane size and potential robustness to high sound-levels, external vibrations, and sudden pressure changes. Ultimately, the squeeze-film microphone enables further down-scaling of microphone technology by at least an order of magnitude thus providing a sound sensing technology that, if brought to higher maturity level, has the potential to complement or partly replace current microphones.

## Supporting Information Available

Fabrication and characterization, spectrogram recorded with the graphene squeeze-film microphone when excited directly with a piezo-electric transducer, transfer function reference microphone and speaker, experimental signal-to-noise ratio and total harmonic distortion, and thermo-mechanical limit of the signal-to-noise ratio (PDF)
