# Supplementary material for: The Graphene Squeeze-Film Microphone
Source: Nano Lett. 2024 Nov 4;24(45):14162–7. doi: 10.1021/acs.nanolett.4c02803 (PMC11566110; doi:10.1021/acs.nanolett.4c02803)
Supplement: Supplementary file 1 — nl4c02803_si_001.pdf [file nl4c02803_si_001.pdf]

The graphene squeeze-film microphone - Supporting Information

Marnix P. Abrahams,[1] Jorge Martinez,[2] Peter G. Steeneken,[1] and Gerard J. Verbiest[1,*]

[1]*Department of Precision and Microsystems Engineering, Delft University of Technology, Mekelweg 2, 2628 CD Delft, The Netherlands, EU*

[2]*Multimedia Computing Group, Intelligent Systems Department, Faculty of Electrical Engineering, Mathematics and Computer Science, Delft University of Technology, 2628 XE Delft, The Netherlands, EU*

1. Fabrication and characterization

A Si wafer with 285 nm dry SiO_2 is spin coated with positive e-beam resist and exposed by electron-beam lithography. Afterwards, the SiO_2 layer without protection is completely etched using CHF_3 and Ar plasma in a reactive ion etcher. The edges of dumbbells are examined to be well-defined by scanning electron microscopy (SEM) and AFM. After resist removal, 2D nanoflakes are exfoliated by Scotch tape, and then separately transferred onto the substrate at room temperature through a deterministic dry stamping technique, as detailed in our earlier work[1]. Using tapping mode atomic force microscopy (AFM), we measure the height difference between the membrane and the Si/SiO_2 substrate. As Fig. S1 shows, we find a membrane thickness t of 18 nm for device 6.

2. Transfer function reference microphone and speaker

The response of the graphene membranes and the reference microphone (AKG C417-L), for example in Fig. 2c of the main text, are convoluted with the transfer function of the speaker (Jabra speak 510). Neither the transfer function of the reference microphone and the speaker are not in detail available, as they also depend on the exact placement with respect to each other. Therefore, we used a well-calibrated Genelec 8020 GPM speaker to estimate the transfer functions. We performed measurements with the Genelec 8020 GPM speaker placed on its tripod and the Jabra speak 510 suspended in mid-air using thin wires. The microphone is also suspended in mid-air using thin wires and is placed at the same distances from the speaker as during the graphene measurements. All devices are attached to a FireFace UFX+ soundcard for the read-out and actuation. We use a frequency sine sweep method[2] to actuate the speakers and measure with the microphone. As the transfer function of the Genelec 8020 GPM speaker is known, we determine the transfer function of the AKG C417-L. In turn, this allows us to determine the transfer function of

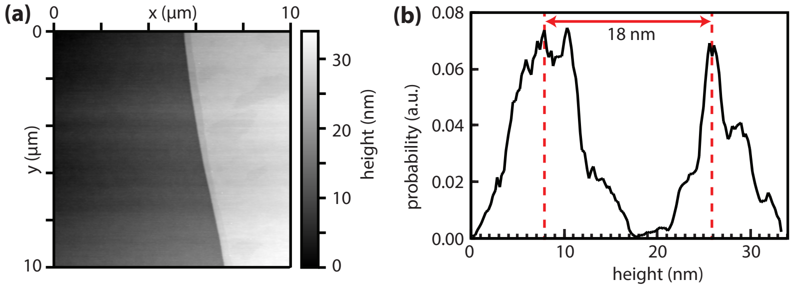

(c)

Device	SNR (dBA)	THD (%)	thickness (nm)	fres (MHz)	Q-factor ()
1	23	12	10	28	1,5
2	20	27	7	20	2,3
3	20	23	9	24	2,7
4	19	21	27	28	4,3
5	14	38	9	22	1,7
6	11	36	18	15	2,8
7	-4	126	6	51	5,4

FIG. S1. (a) Atomic force microscopy (AFM) topography image of graphene membrane on device 6. (b) normalized height distribution of the AFM image shown in panel (a). The dashed red lines indicate the center of the peaks corresponding to the surface of the substrate and of the membrane. The distance of 18 nm between the dashed red lines corresponds to the thickness of the membrane. (c) table of the determined signal-to-noise ratio (SNR), total harmonic distortion (THD), thickness (nm), resonance frequency (MHz), and Q-factor of the measured devices.

* Corresponding author: G.J.Verbiest@tudelft.nl

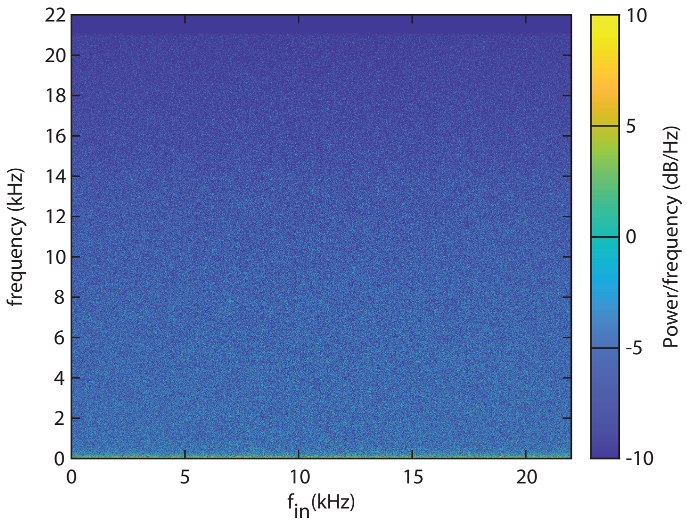

FIG. S2. Spectrogram recorded with the graphene squeeze-film microphone as described in the main text when glued upon and directly excited with a piezo-electric transducer. The absence of a signal indicates the graphene squeeze-film microphone is insensitive to sound and vibrations coupling in trhrough the substrate.

the Jabra speak 510. This information is used to deconvolute the speaker response from the graphene response in Fig. 2c and 2d.

3. Experimental signal-to-noise ratio and total harmonic distortion

We determined the signal-to-noise ratio and total harmonic distortion for all graphene devices. Following state-of-the-art procedures for MEMS microphones, we specify the signal-to-noise ratio for an acoustic excitation of 80 dB_{SPL} at 1 kHz. First, we make a spectrogram of the data recorded from the reference microphone and the graphene device while sweeping f_{in} from 20 Hz to 22 kHz. Using the spectrogram of the reference microphone, we create a mask for the signal at f_{in} at 1 kHz and at n integer multiples of f_{in}. The mask at f_{in} is used to identify the signal in the spectrogram of the graphene device and the ones at nf_{in} to determine the total harmonic distortion. We then select a small band around $f_{in} = 1$ kHz of 350 Hz that is not affected by the phase lock loss of the PLL and is thus representative for the signal. We repeat this procedure for small bands around n integer multiples of f_{in}, which is thus representative for the total harmonic distortion. The remaining parts of the spectrogram of the graphene device represent the left-over noise. Then, we make an inverse Fourier transform of the identified signal, total harmonic distortion, and the left-over noise. Utilising the time domain results we acquire the signal, total harmonic distortion, and noise powers in dBA, in accordance with the theory as stated in[3,4] and using the Matlab code which can be found at[5]. The resulting signal, total harmonic distortion, and noise powers and then averaged to find a global value for the entire measurement. Finally, we subtract the signal and noise powers to acquire the signal-to-noise ratio. We find the total harmonic distortion by the total harmonic distortion power by the sum of the total harmonic distortion power and the signal power and then convert this number from dBA into a percentage.

4. Thermo-mechanical limit of the signal-to-noise ratio

The squeeze-film effect relates the resonance frequency f_r of the graphene membrane to the ambient pressure P_{amb} and the distance g_0 to the back-plate:

$$f_{res}^2 = f_0^2 + \frac{P_{amb}}{4\pi^2 g_0 \rho h}. \tag{S1}$$

Here, ρ is the mass density of the graphene and h its thickness. It is important to realize that the motion $\delta x(t)$ as a function of time t of the graphene membrane itself also modulates the distance g_0. The true distance between the

graphene and the back-plate is given by $g_0 + \delta x(t)$. According to equation S1, this will result in frequency fluctuation and thereby set a limit on the performance of squeeze-film microphones. We can estimate this limit by making a Taylor expansion of equation S1 in $\delta x(t)$:

$$f_{\rm res}^2 + 2f_{\rm res}\delta f(t) + \delta f^2 = f_0^2 + 2f_0\delta f_0(t) + \delta f_0^2(t) + \frac{P_{\rm amb}}{4\pi^2 g_0 \rho h}\left(1 - \frac{\delta x(t)}{g_0} + \frac{\delta x(t)^2}{g_0^2}\right). \tag{S2}$$

In this expansion, we already included the frequency fluctuations $\delta f_{\rm res}(t)$ and $\delta f_0(t)$. The former represents the frequency fluctuations of the squeeze-film microphone and the latter the frequency fluctuations in vacuum conditions.

In order to quantify the resulting frequency fluctuations $\delta f_{\rm res}(t)$, we take the time average of equation S2 and subtract equation S1 to find:

$$< \delta f^2 > = < \delta f_0^2(t) > + \frac{P_{\rm amb}}{4\pi^2 g_0 \rho h}\frac{< \delta x(t)^2 >}{g_0^2}. \tag{S3}$$

Here, $< ... >$ denotes a time-averaged value. In this step, we assumed that $< x(t) >$, $< \delta f_{\rm res}(t) >$ and $< \delta f_0(t) >$ are zero.

From equipartition theorem, we find an analytical expression for $< \delta x(t)^2 >$:

$$< \delta x(t)^2 > = \frac{k_{\rm B}T}{4\pi^2 m_{\rm eff} f_{\rm res}^2}, \tag{S4}$$

where the mass $m_{\rm eff}$ is the effective mass of the fundamental mode of the membrane and is given by $m_{\rm eff} = \gamma \rho h \pi r^2$, in which $\gamma = 0.269$. We denote the radius of the membrane with r.

By inserting equation S4 in equation S3 and setting $< \delta f_0^2(t) >$ equal to zero, we find a fundamental limit for the frequency fluctuations $< \delta f^2 >$ of a squeeze-film microphone:

$$< \delta f^2 > = \frac{P_{\rm amb} k_{\rm B} T \gamma \pi r^2}{16\pi^4 g_0^3 m_{\rm eff}^2 f_{\rm res}^2}. \tag{S5}$$

In order to estimate the ultimate signal-to-noise ratio of squeeze-film microphone, we also need the sensitivity S:

$$S = \frac{\partial f_{\rm res}}{\partial P_{\rm amb}} = \frac{1}{8\pi^2 g_0 \rho h f_{\rm res}} = \frac{\gamma \pi r^2 f_{\rm res}}{2kg_0}. \tag{S6}$$

The second equality in Eq. S6 arises from $2\pi f_{\rm res} = \sqrt{k/m_{\rm eff}}$, in which $1/k$ is the compliance of the membrane. For commercial microphones, the signal-to-noise ratio is commonly specified at a sound pressure level of 80 $\rm dB_{SPL}$, which corresponds to a pressure wave with an amplitude of 0.2 Pa. The signal one expects from the squeeze-film microphone is then $0.2S$. When denoting the noise source by N, we find a SNR ratio of:

$$SNR_{\rm limit} = 20\log\left(\frac{0.2S}{N}\right) = 20\log\left(\frac{0.2\gamma\pi r^2 f_{\rm res}}{2kg_0 N}\right). \tag{S7}$$

In case N is set by the thermo-mechanical noise, we find the ultimate noise limit is given by $\sqrt{< \delta f^2 >}$. This leads to the following signal-to-noise ratio in dB:

$$SNR_{\rm limit} = 20\log\left(\frac{0.2S}{\sqrt{< \delta f^2 >}}\right) = 20\log\left(\frac{0.1\sqrt{\gamma\pi r^2 g_0}}{\sqrt{P_{\rm amb} k_{\rm B} T}}\right). \tag{S8}$$

The ultimate signal-to-noise ratio solely depends on the radius of the membrane, the ambient pressure, and the temperature.

References

[1] H. Liu, S. Basuvalingam, S. Lodha, A. Bol, H. van der Zant, P. Steeneken, and G. Verbiest, Nanomechanical resonators fabricated by atomic layer deposition on suspended 2d materials, 2D Materials **10**, 045023 (2023).
[2] S. Orlando, B. Peeters, and G. Coppotelli, Improved frf estimators for mimo sine sweep data., Proceedings of the ISMA 2008 International Conference on Noise and Vibration Engineering , 229 (2008).
[3] H. Fletcher and W. Munson, Loudness, its definition, measurement and calculation, J. Acoust. Soc. Am. , 82 (1933).
[4] A. T. S. for Sound Level Meters Z24.3-1936 for Measurement of Noise and O. Sounds, J. Acoust. Soc. Am. , 147 (1936).
[5] Mathworks (C. Couvreur), https://nl.mathworks.com/matlabcentral/fileexchange/69-octave (Accessed 2024-09-24).
